# Small GTPase Rab7-mediated FgAtg9 trafficking is essential for autophagy-dependent development and pathogenicity in *Fusarium graminearum*

**DOI:** 10.1371/journal.pgen.1007546

**Published:** 2018-07-25

**Authors:** Huawei Zheng, Pengfei Miao, Xiaolian Lin, Lingping Li, Congxian Wu, Xiaomin Chen, Yakubu Saddeeq Abubakar, Justice Norvienyeku, Guangpu Li, Jie Zhou, Zonghua Wang, Wenhui Zheng

**Affiliations:** 1 State Key Laboratory of Ecological Pest Control for Fujian and Taiwan Crops, College of Plant Protection, Fujian Agriculture and Forestry University, Fuzhou, China; 2 Fujian University Key Laboratory for Plant-Microbe Interaction, College of Life Sciences, Fujian Agriculture and Forestry University, Fuzhou, China; 3 Department of Biochemistry and Molecular Biology, University of Oklahoma Health Sciences Center, Oklahoma City, United States of America; 4 Institute of Oceanography, Minjiang University, Fuzhou, China; Oregon State University, UNITED STATES

## Abstract

*Fusarium graminearum* is a fungal pathogen that causes Fusarium head blight (FHB) in wheat and barley. Autophagy is a highly conserved vacuolar degradation pathway essential for cellular homeostasis in which Atg9 serves as a multispanning membrane protein important for generating membranes for the formation of phagophore assembly site. However, the mechanism of autophagy or autophagosome formation in phytopathogens awaits further clarifications. In this study, we identified and characterized the Atg9 homolog (FgAtg9) in *F*. *graminearum* by live cell imaging, biochemical and genetic analyses. We find that GFP-FgAtg9 localizes to late endosomes and trans-Golgi network under both nutrient-rich and nitrogen starvation conditions and also show its dynamic actin-dependent trafficking in the cell. Further targeted gene deletion of *FgATG9* demonstrates that it is important for growth, aerial hyphae development, and pathogenicity in *F*. *graminearum*. Furthermore, the deletion mutant (Δ*Fgatg9*) shows severe defects in autophagy and lipid metabolism in response to carbon starvation. Interestingly, small GTPase FgRab7 is found to be required for the dynamic trafficking of FgAtg9, and co-immunoprecipitation (Co-IP) assays show that FgAtg9 associates with FgRab7 *in vivo*. Finally, heterologous complementation assay shows that Atg9 is functionally conserved in *F*. *graminearum* and *Magnaporthe oryzae*. Taken together, we conclude that FgAtg9 is essential for autophagy-dependent development and pathogenicity of *F*. *graminearum*, which may be regulated by the small GTPase FgRab7.

## Author summary

Autophagy is an intracellular degradation pathway conserved in eukaryotes, but the mechanism of autophagy or autophagosome formation in the wheat head blight fungus *Fusarium graminearum* remains unclear. One fundamental question in the autophagy field lies on how the formation of autophagosome and recycling of cellular elements to ensure survival under stress conditions is achieved. Atg9 is the sole multi-spanning membrane protein of the autophagy-related proteins. In this study, we observed the localization pattern of FgAtg9 in *F*. *graminearum* by live cell imaging and demonstrated that it is essential for autophagy, development and pathogenicity in *F*. *graminearum*. Furthermore, we found that the small GTPase FgRab7 is required for FgAtg9 trafficking and FgRab7 associates with FgAtg9 in an *in vivo* Co-IP assay. These results widen our understanding of the relationship bewtween membrane traficking and autophagy-dependent development and pathogenicity of plant fungal pathogens.

## Introduction

Pathogenic fungi are great threats to both plants and animals, hence jeopardizing food security [1]. *Fusarium graminearum* is a plant fungal pathogen which causes head blight of wheat and other cereals and has become a serious problem to agricultural production in the world [2–4]. During Fusarium head blight infection, the fungus forms lobate appressoria and infection cushions which help it gain entry into the host cell, or may enter the cell through vulnerable openings and the stomata, and then colonizes the host cells through hyphal elongation [5,6]. It also produces mycotoxins such as deoxynivalenol (DON) and zearalenone in cereal grains and animal feeds making them unfit for consumption [7,8]. Recent studies suggest that intracellular trafficking including endocytosis, exocytosis, retrograde trafficking and ESCRT pathway are all important for the development, pathogenicity, and production of DON in *F*. *graminearum* [9–14].

In all eukaryotic cells, autophagy is a dynamic process essential for cell homeostasis and involves rearrangement of subcellular membranes to sequester cytoplasm and organelles for delivery to the lysosome or vacuole where the sequestered cargoes are degraded and recycled within the cell for survival during nutrient-starvation [15,16]. Disruption of autophagy causes diseases in mammals, including cancer, liver disease, muscular disorder and neurodegeneration [17]. In fungi, autophagy is typically induced by nutrient-starvation or by the macrolide rapamycin. Upon induction, target of rapamycin kinase is inhibited and a double membrane vesicle sequesters some organelles and the cytosol, forming an autophagosome. The autophagosome subsequently docks with the vacuole and fuses with the vacuolar membrane. In this process, the autophagic substrates are degraded by vacuolar proteases and recycled [18]. In yeast, more than 30 genes have been originally identified to be involved in various steps of autophagy [19–21]. Seventeen autophagy proteins (Atg) are commonly required for core autophagic machinery, whereas another sixteen proteins have more specific roles [22]. Atg8 is a core component of the ubiquitin-like protein conjugation systems that are essential for autophagosome formation [23].

Autophagosome is a large cytosolic double-membrane vesicle for degradation of sequestered autophagic cargoes [15,16]. The successive fusions of autophagosomes with yeast or fungal vacuole, deliver luminal cargoes for degradation by resident hydrolases. Genetic screens in yeast led to the isolation of most of the known components specifically involved in autophagosome biogenesis [21]. In yeast, the autophagosome originates at a precise and unique location in the cell called the pre-autophagosomal structure or phagophore assembly site (PAS). PAS is not a stable organelle, it is rather an autophagosomal intermediate in continuous sequential disappearance and reformation [20]. Various organelles including the Golgi complex, endoplasmic reticulum, plasma membrane, endosomes and mitochondria might act as a membrane source for autophagosome formation [22,24]. Autophagy is a multistep process, and different Atg proteins are used sequentially for subsequent completion of the process.

Autophagy has been studied in several pathogenic fungi [23], including *Magnaporthe oryza*, *Colletotrichum*, *Ustilago* and *Fusarium*. Genome-wide functional analysis reveals that infection-associated fungal autophagy is necessary for the development of rice blast disease [25–27]. Autophagy contributes to regulation of nuclear dynamics during vegetative growth and hyphal fusion in *Fusarium oxysporum* [28]. Recently, Lv et al. reported that FgAtg1- and FgAtg5- mediated autophagy are necessary for the development and virulence of *F*. *graminearum* [29]. Previous studies showed that FgAtg8 provides nutrients for nonassimilating fungal structures and is necessary for plant colonization in *F*. *graminearum* [30]. FgAtg15 is important for lipid turnover and plant infection [31]. However, the mechanism of autophagy and/or autophagosome formation is still unclear in *F*. *graminearum* and many other plant pathogens.

Atg9 is the only integral membrane component of the conserved Atg machinery and functions in delivering membranes to the expanding phagophore for autophagosome formation [20,22]. In yeast, Atg9 is transported from the Golgi to the PAS and/or early autophagosomal precursors in small, highly motile vesicles and then retrieved from complete autophagosomes and/or vacuole membranes. Atg9 cannot be retrieved from the PAS in the absence of Atg1 [32]. Phosphorylation of Atg9 by Atg1 is required for phagophore formation [33]. Atg9 is not exclusively localized to the pre-autophagosomal structure, but also distributed in several cytoplasmic punctate structures [20]. In mammalian cells, Atg9 localizes to the trans-Golgi network (TGN) and endosomes under nutrient-rich conditions, whereas it translocates to autophagosomes under starvation conditions [34]. Sec2, Sec4, Atg23, Atg27, and the actin cytoskeleton are known to participate in anterograde delivery of Atg9 to the PAS, whereas Atg1, Atg13, Atg2, Atg18, and the phosphatidylinositol (PtdIns) 3-kinase Vps34 are required for its retrograde movement [23,35,36]. Numerous Rab GTPases have been shown to be involved in various stages of autophagy [37]. For example, Rab1, Rab5, Rab7, Rab9A, Rab11, Rab23, Rab32, and Rab33B play an important role in autophagosome formation. Furthermore, Rab1 and Rab11 were reported to be important for both proper Atg9A localization and autophagosome formation in mammalian cells [38,39]. Our recent study demonstrated that Rab GTPases are essential for membrane trafficking in *F*. *graminearum* [12], and they may regulate the anterograde and/or retrograde trafficking of Atg9 in this pathogenic fungus.

In this study, we generated null mutants of *FgATG9* and systematically studied its function in autophagosome formation, fungal development and its trafficking mechanism in the cell. Live cell imaging shows that FgAtg9 localizes to the late endosomes and TGN. We have also shown that the trafficking of FgAtg9 depends on the actin cytoskeleton. Genetic and biochemical analyses demonstrate that FgAtg9 is important for the formation of autophagosome, aerial hyphae development, and pathogenicity in *F*. *graminearum*. Furthermore, we found that FgRab7 is required for the trafficking of GFP-FgAtg9.

## Results

### Identification of the autophagy-related gene *FgATG9* in *Fusarium graminearum*

Using the *S*. *cerevisiae* Atg9 amino acid sequence as a trace to blast the available fungal genome database, we identified an Atg9 homologue at the FGSG_13660 locus. FGSG_13660 is predicted to encode a 901-amino-acid protein that shares 39% identity with *S*. *cerevisiae* Atg9 and 49% identity with *M*. *oryzae* Atg9, and is named here as FgAtg9. Atg9 is a multispanning membrane protein and is required for generating membranes for the formation of PAS [20]. Further domain analysis revealed that FgAtg9 possesses five transmembrane domains (S1A Fig), 210–232 aa, 265–287 aa, 436–458 aa, 521–543 aa, and 559–578 aa, as similar to five transmembrane domains of MoAtg9 in *M*. *oryzae*, contrary to six transmembrane domains in yeast and *F*. *oxysporum* (S1A Fig). Phylogenetic anslysis of FgAtg9 and other Atg9 proteins showed the presence of a single gene in filamentous fungi, but two isoforms in mammals (S1B Fig). These data suggest that Atg9 homologs are highly conserved in fungi.

### The subcellular localization of FgAtg9 in *F*. *graminearum*

**Fig 1:**
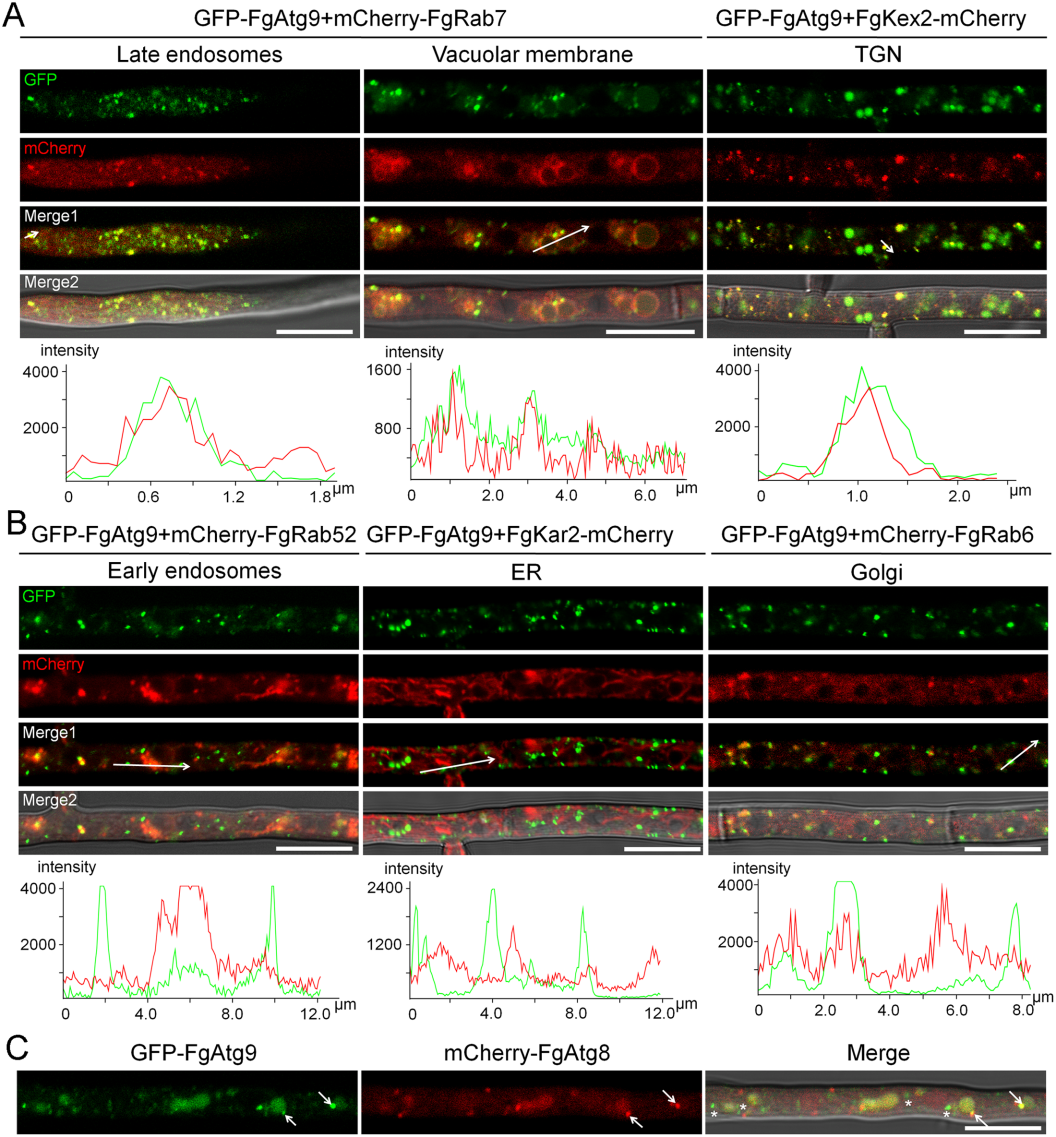
The subcellular localization of FgAtg9 in nutrient-rich condition. (A-B) Micrographs and their corresponding line scan graphs showing the localization of GFP-FgAtg9–labelled vesicles with early endosomal (mCherry-FgRab52), late endosomal or vacuolar membrane (mCherry-FgRab7), ER (FgKar2-mCherry), TGN (trans-Golgi network, FgKex2-mCherry), and Golgi (mCherry-FgRab6) markers in growing hyphae of *F*. *graminearum* in nutrient-rich medium (CM). Bar = 10 μm. (C) GFP-FgAtg9 partially co-localized with mCherry-FgAtg8 in nutrient-rich medium. Arrows show co-localiztion, asterisks indicate non-co-localization. Bar = 10 μm.

To determine the subcellular localization of FgAtg9 in *F*. *graminearum*, a GFP sequence was fused to the N-terminus of FgAtg9 using ToxA promoter which effectively expressed in *F*. *graminearum* [12]. We found that GFP-FgAtg9 localizes to punctate structures and displays dynamic mobility with uneven distribution in mycelial cytoplasm (Fig 1; S2 Fig; S1 Video). The protein was also observed to be expressed at different stages of conidial development (0 h, 4 h, 8 h; S2 Fig). To investigate whether the movement of GFP-FgAtg9 is dependent on microtubules and/or the actin cytoskeletons, we treated freshly harvested mycelia with Latrunculin A (an actin cytoskeleton inhibitor) and Nocodazole (a microtubule-destabilizing agent), respectively [9], using DMSO treatment as control (S2 Video; S3 Fig). We found that the dynamic movement of GFP-FgAtg9 became much slower (S3 Video; S3 Fig) when treated with Latrunculin A. By contrast, the trafficking of GFP-FgAtg9 was not significantly affected when treated with Nocodazole (S4 Video; S3 Fig). Taken together, these results suggest that the FgAtg9 trafficking requires actin cytoskeleton.

**Fig 2:**
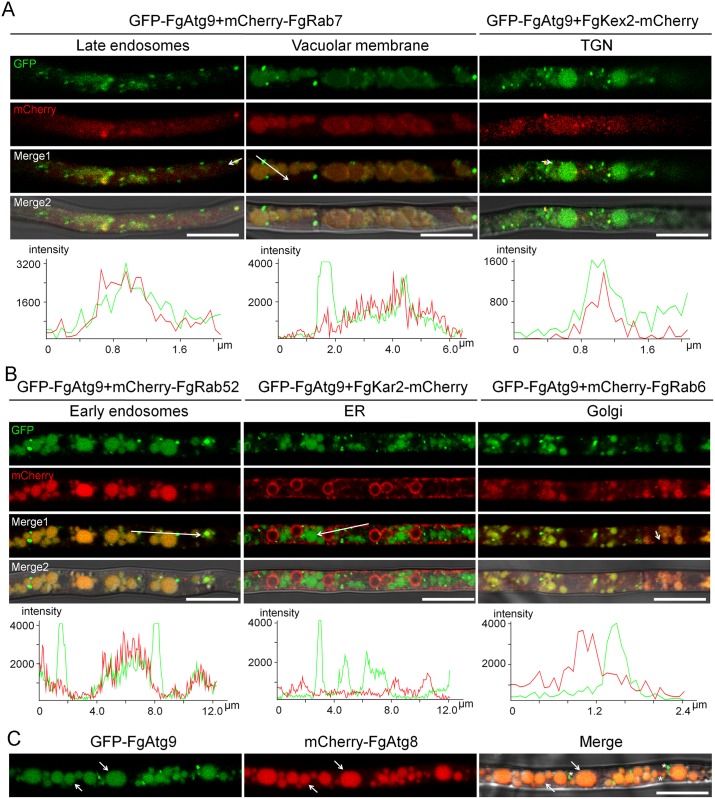
The subcellular localization of FgAtg9 in nitrogen starvation condition. (A-B) Micrographs and their corresponding line scan graphs showing the localization of GFP-FgAtg9–labelled vesicles with early endosomal, late endosomal or vacuolar membrane, ER, TGN, and Golgi markers in *F*. *graminearum* grown under nitrogen starvation medium (MM-N). Bar = 10 μm. (C) GFP-FgAtg9 partially co-localized with mCherry-FgAtg8 under nitrogen starvation. Arrows indicate co-localiztion whereas asterisks show non-co-localization. Bar = 10 μm.

Since the precise localization of Atg9 in plant pathogens is not well known, we next investigated the nature of the GFP-FgAtg9-containing punctate structures in the cell by co-transforming GFP-FgAtg9 respectively with the early endosomal marker mCherry-FgRab52, late endosomal and vacuolar membrane marker mCherry-FgRab7, ER marker FgKar2-mCherry, medial Golgi marker mCherry-FgRab6, and TGN marker FgKex2-mCherry [12], into the protoplast of the wild-type strain (PH-1), and examined their intracellular localization by fluorescence microscopy. We found that GFP-FgAtg9 partially colocalized with FgRab7-positive late endosomes (57.11±7.95% colocalization) and FgKex2-positive TGN (54.42±11.70% colocalization) (Fig 1A) in nutrient-rich CM medium, and closely associated with vacuolar membrane (Fig 1A). However, FgAtg9 showed no obvious co-localization with the early endosomal, ER and medial Golgi markers in CM medium (Fig 1B). Under nitrogen starvation (MM-N medium), we found that most of the GFP-FgAtg9 signals were translocated to the vacuole/autophagosome (Fig 2), and the punctate vesicles cycled between the cytoplasm and vacuole/autophagosome (S5 Video). Consistently, FgAtg9 also partially colocalized with the late endosomes (42.17±12.24% colocalization), TGN (30.94±8.25% colocalization), and was closely associated with vacuolar membrane (Fig 2A), but no obvious co-localization with the early endosomes, ER and medial Golgi (Fig 2B). Taken together, these results suggest that FgAtg9 mainly localizes in the late endosomes and TGN of *F*. *graminearum*.

Atg9 is proposed to mediate membrane transport to generate autophagosomes in mammalian cells [40]. The ubiquitin-like Atg8 has been shown to be essential for autophagosome formation and is often used as a biological marker for tracking the autophagy process as it is associated with all stages of the process of autophagy [41]. When GFP-FgAtg9 was co-transformed with mCherry-FgAtg8 (a marker gene for autophagy), we observed that FgAtg9 only partially colocalized with the mCherry-FgAtg8 in both CM nutrient-rich and MM-N media (Figs 1C and 2C), suggesting that FgAtg9 not only collaborates with FgAtg8, but also has distinct functions in *F*. *graminearum*.

### Generation and characterization of *FgATG9* gene deletion mutant

In order to study the function of FgAtg9, we generated deletion mutants by replacing *FgATG9* gene with hygromycin phosphotransferase (*hph*) gene as the selectable marker in the wild-type strain (PH-1) (S4A Fig), and identified four *FgATG9* deletion transformants by PCR. The gene deletion transformants Δ*Fgatg9-1*, Δ*Fgatg9-2*, and Δ*Fgatg9-3* were confirmed by Southern blot analysis, which showed a 4.46 kb band in the PH-1 and a 2.87 kb band in the mutants (S4B Fig). Furthermore, the *FgATG9* gene with its native promoter was reintroduced into the protoplast of Δ*Fgatg9-2*, resulting in the complemented strain Δ*Fgatg9-C* confirmed by Southern blot (S4B Fig). The PH-1, Δ*Fgatg9-2*, Δ*Fgatg9-3*, and Δ*Fgatg9-C* strains were used for further phenotype analyses.

### Autophagy is blocked in the *FgATG9* deletion mutants

**Fig 3:**
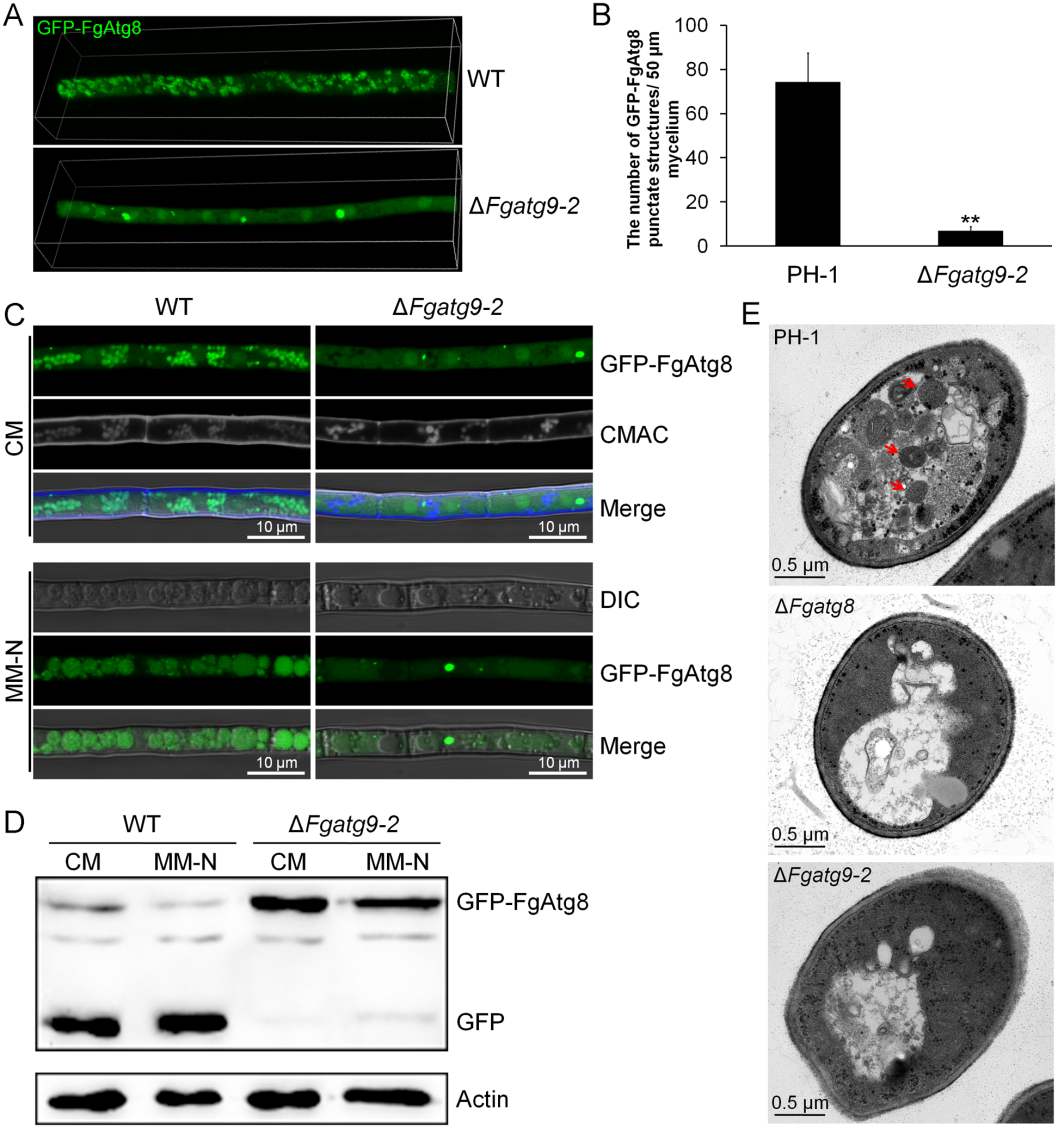
FgAtg9 is required for autophagosome formation and autophagy in *F*. *graminearum*. (A) 3D (three-dimensional) micrographs showing different expression and localization of GFP-FgAtg8 (autophagosome marker) in PH-1 and Δ*Fgatg9* mutant using a laser confocal microscope. Width, 106.07 μm; height, 26.52 μm; depth, 11.60 μm. (B) Quantitative analysis of the number of GFP-FgAtg8-labelled vesicles of 50 μm hyphae in PH-1 and Δ*Fgatg9* mutant. The mean ± SD were calculated based on three independent experiments and double asterisks represent significant differences at p < 0.01 according to *t*-test. (C) Localization of GFP-FgAtg8 in PH-1 and Δ*Fgatg9* mutant in CM or MM-N media. PH-1 and Δ*Fgatg9* mutant expressing GFP-FgAtg8 were grown in liquid CM medium at 28 °C for 16 h and then shifted to liquid MM-N medium with 2 mM PMSF for 8 h to induce autophagy. Mycelia were stained with CMAC and examined using confocal microscope. Bar = 10 μm. (D) Proteolysis assays of GFP-FgAtg8 in PH-1 and Δ*Fgatg9* mutant. Mycelia cultured at 28 °C for 16 h in CM liquid medium were continuously shaken at 150 rpm. Autophagy was induced after 8 h of nitrogen starvation with 2 mM PMSF. Mycelia were collected and extracted for Western blot using anti-GFP, and anti-actin was shown as a loading control. (E) Observation of autophagosome in the hyphal vacuoles of the PH-1, Δ*Fgatg8* and Δ*Fgatg9* mutants by transmission electron microscopy. Wild type PH-1, Δ*Fgatg9* and Δ*Fgatg*8 strains were cultured in liquid CM medium at 28 °C for 16 h, and then shifted to liquid MM-N medium with 2 mM PMSF for 8 h. The vacuoles of PH-1 hyphal cells were filled with autophagosomes, while no autophagosomes were found inside the vacuoles of Δ*Fgatg8* and Δ*Fgatg9* mutants. Red arrows indicate the autophagic bodies.

After induction of autophagy, GFP-Atg8 is transported into the vacuole where the GFP moiety is released by proteolysis and is relatively stable, thereby reflecting the level of autophagy [42]. It was reported that Atg8 localization to the PAS is dependent on the presence of Atg9 [43], so we introduced GFP-FgAtg8 into the PH-1 and Δ*Fgatg9* mutant, respectively, and found that GFP-FgAtg8 was localized to punctate structures throughout the cytoplasm in the CM medium of the wild type (WT) (Fig 3A). However, the GFP-FgAtg8-containing punctate structures were significantly reduced in the Δ*Fgatg9* mutant as seen from 3D (three-dimensional) micrographs (Fig 3A and 3B). Furthermore, we used CMAC to stain the vacuole and we found numerous autophagic bodies (GFP-FgAtg8-containing punctate structures) in the vacuoles of WT (Fig 3C), but not in the vacuoles of Δ*Fgatg9* mutant (Fig 3C). Under nitrogen starvation (MM-N medium) condition, GFP-FgAtg8 was transported into and accumulated in the vacuoles of WT while its localization remained in the cytoplasm of the Δ*Fgatg9* mutant (Fig 3C), suggesting a block of FgAtg8 trafficking to the vacuole in the Δ*Fgatg9* mutant. To further substantiate our observation, GFP-FgAtg8 proteolysis assay was performed. Under the nutrient-rich conditions, a full-length GFP-FgAtg8 band (40 kDa) and a GFP band (26 kDa) were detected in the PH-1 with an anti-GFP antibody (Fig 3D). When the hyphae were shifted to MM-N conditions, GFP-FgAtg8 proteolysis was more robust (Fig 3D). By contrast, GFP-FgAtg8 proteolysis was significantly blocked in the Δ*Fgatg9* mutant under both nutrient-rich and MM-N conditions. These results indicate that GFP-FgAtg8 proteolysis, a hallmark of autophagy is defective in the Δ*Fgatg9* mutant.

Next, transmission electron microscopy was used to further investigate the autophagic bodies of the wild type PH-1, Δ*Fgatg8* and Δ*Fgatg9* mutants. Consistently, little autophagic bodies were seen in the vacuoles of both Δ*Fgatg8* (negative control) and Δ*Fgatg9* mutants (Fig 3E). By contrast, autophagic bodies were abundant and clearly visible in the vacuoles of PH-1 (Fig 3E). These results further demonstrate that autophagy is blocked in the *FgATG9* deletion mutant.

### FgRab7 is required for FgAtg9 trafficking

**Fig 4:**
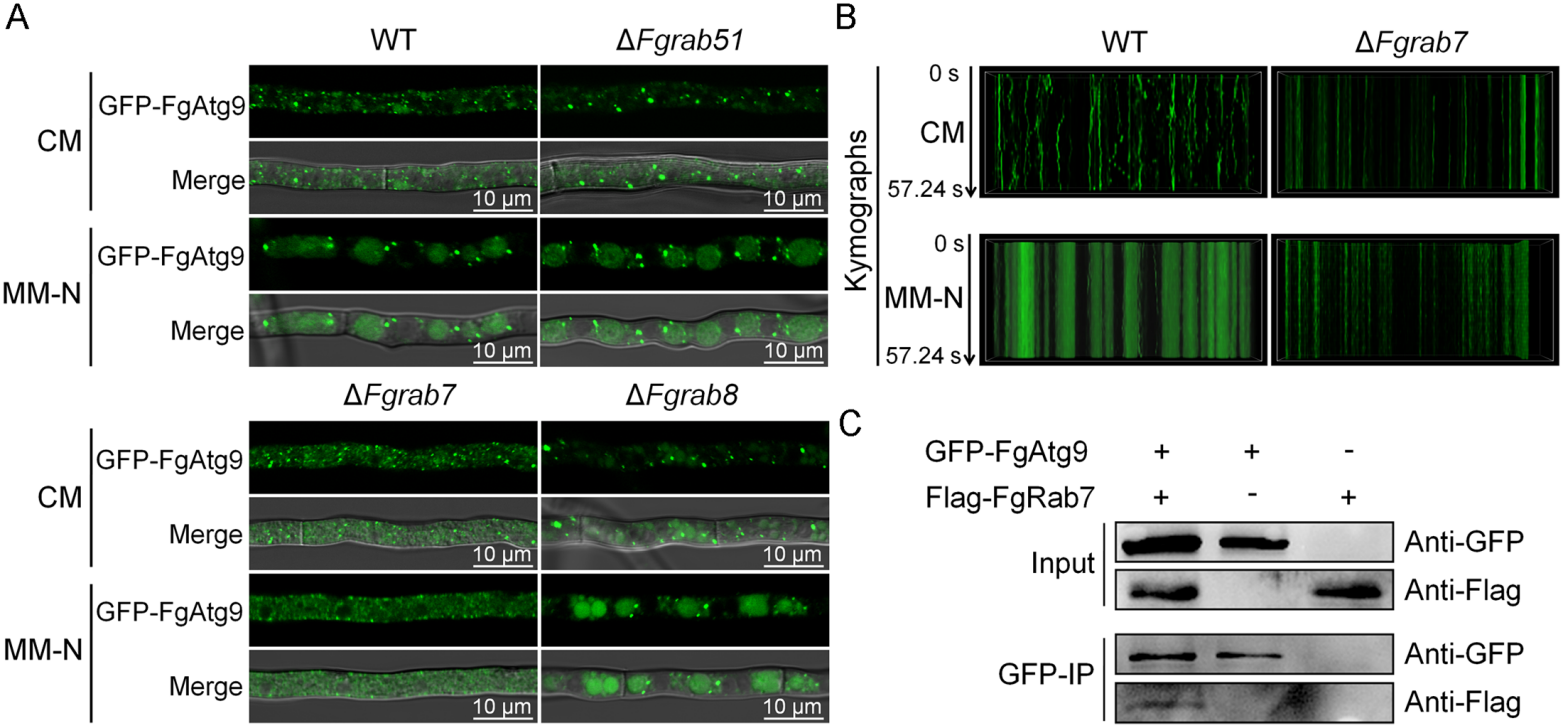
FgRab7 is required for FgAtg9 trafficking. (A) *FgRAB51*, *FgRAB7* and *FgRAB8* deletion mutants expressing the GFP-FgAtg9 fusion protein were inoculated in CM and MM-N conditions with 2 mM PMSF to observe FgAtg9 trafficking. GFP-FgAtg9 appeared diffused in the cytosol of Δ*Fgrab7* mutant compared with PH-1. (B) Kymographs of “stream” time-lapse series of GFP-FgAtg9 in PH-1 and Δ*Fgrab7* in CM and MM-N conditions evidenced from S1, S5, S6 and S7 Videos respectively. The GFP-FgAtg9 punctate compartments were either static or showed highly restricted movements in Δ*Fgrab7* mutant in comparison with PH-1 under both CM and MM-N conditions. X-axis indicates the length of hyphae. Y-axis represents the time. All kymographs with time and length dimensions indicated are displayed at the same scale and directly comparable. Time duration is 57.24 s. (C) GFP-trap-based pull-down experiment indicating the interaction between the GFP-FgAtg9 and Flag-FgRab7 in *F*. *graminearum*.

We have previously demonstrated that Rab GTPases are essential for membrane trafficking in *F*. *graminearum* [12] and they have been reported to play important roles in regulating autophagy [37,44]. Thus one or more FgRab GTPases may play a role in regulating the trafficking of FgAtg9 during cell autophagy. To test this hypothesis, we transformed GFP-FgAtg9 expression construct into the *FgRAB51*, *FgRAB7*, and *FgRAB8* deletion mutants. The resulting transformants were confirmed by polymerase chain reaction (PCR) and screened by GFP signal, then examined for localization and intracellular trafficking of GFP-FgAtg9 in these mutants by live cell imaging. We found that GFP-FgAtg9 displayed punctate localization similar to that observed in the wild type under nutrient-rich conditions (Fig 4A). However, we found that the dynamic mobility and trafficking of GFP-FgAtg9 in *FgRAB7* deletion mutant was much slower or almost static in vegetative mycelia (Fig 4B; S6 Video). Consistently, GFP-FgAtg9 also appeared more diffused or static in the cytosol of the *FgRAB7* deletion mutant under nitrogen starvation condition (Fig 4A and 4B; S7 Video), while GFP-FgAtg9 punctate vesicles were closely associated with vacuolar/autophagosome membrane or the cytoplasm in the vegetative mycelia of the wild type, and cycled between the vacuole/autophagosome and cytoplasm (Fig 4A and 4B; S5 Video). The kymograph further confirmed that the dynamics of GFP-FgAtg9 in Δ*Fgrab7* mutant are slower than in the wild type (Fig 4B). To further determine the relationship of FgAtg9 with FgRab7, we investigated whether FgAtg9 could associate with FgRab7 *in vivo*. In co-immunoprecipitation (Co-IP) assays with transformants expressing GFP-FgAtg9 and Flag-FgRab7 constructs, Flag-FgRab7 fusion proteins could be detected in proteins co-purified with GFP-FgAtg9 using anti-GFP beads (Fig 4C). Taken together, these results show that FgRab7 is required for FgAtg9 trafficking in the cells.

### FgAtg9 is required for vegetative growth and aerial hyphae development

**Fig 5:**
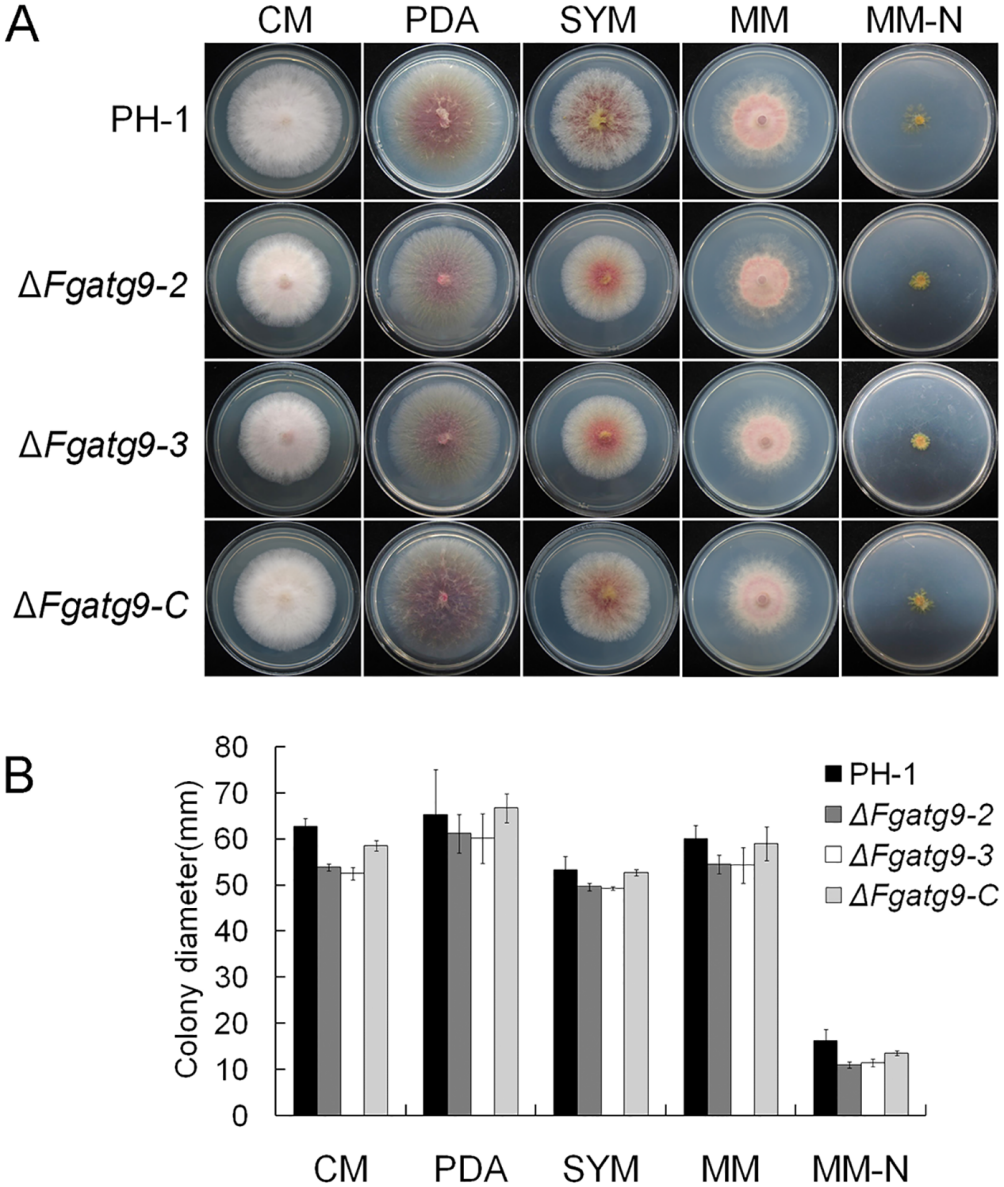
Colony morphology and growth of Δ*Fgatg9* mutants. (A) Colony morphology of the wild type (PH-1), *FgATG9* deletion mutants (Δ*Fgatg9-2*, Δ*Fgatg9-3*) and complemented strain (Δ*Fgatg9-C*) grown on CM, PDA, SYM, MM and MM-N agar for 3 days. (B) Colony diameter of Δ*Fgatg9* mutants on CM, PDA, SYM, MM, MM-N agar after 3 days.

**Fig 6:**
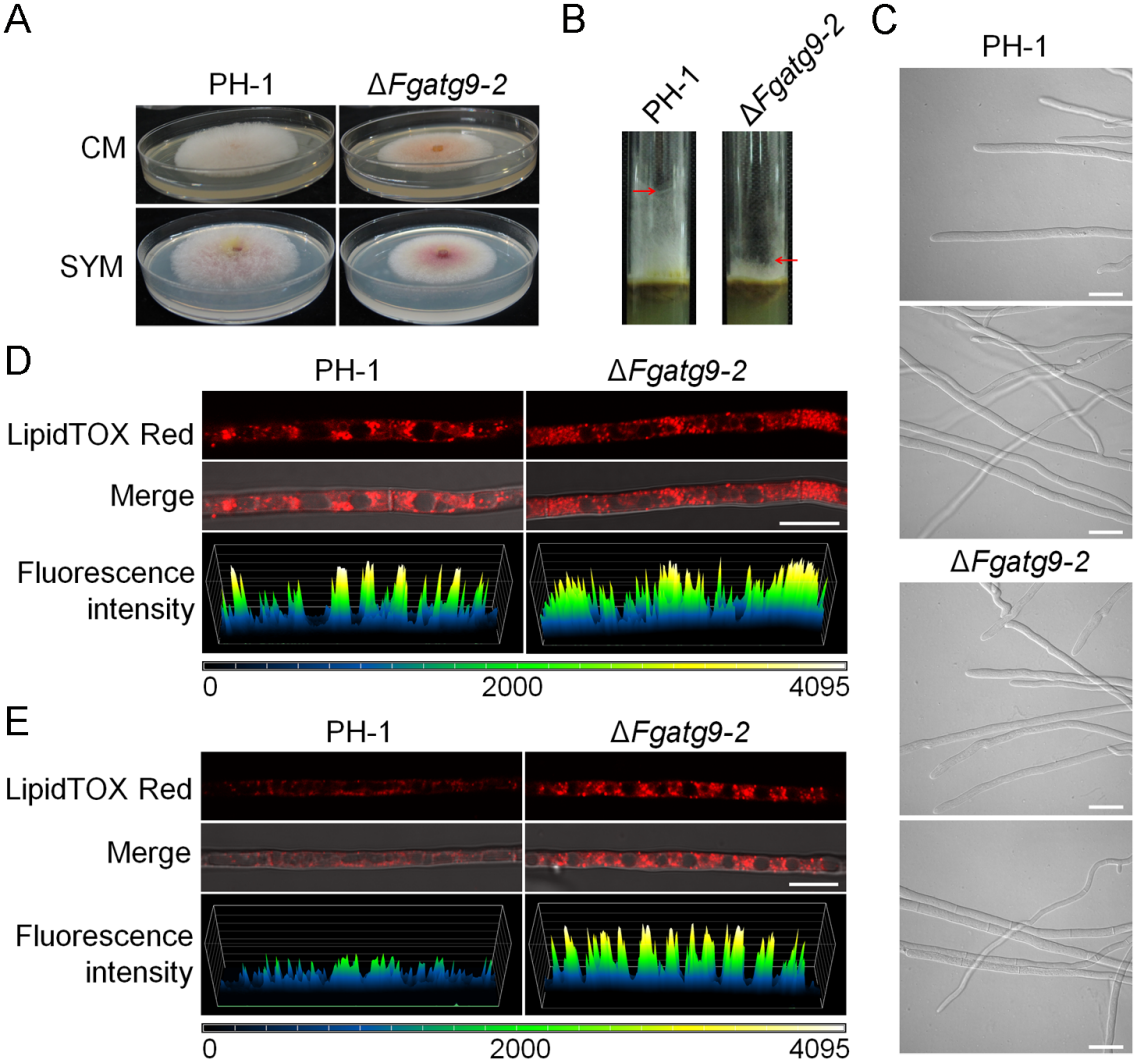
Aerial hyphae growth defects and reduction in lipid droplet degradation of *FgATG9* deletion mutant. (A) The aerial hyphae morphology of Δ*Fgatg9* mutant in CM and SYM agar plates. (B) Aerial hyphae morphology of Δ*Fgatg9* mutant in glass tube containing CM agar. (C) Microscopic observation of the hyphae of wild type PH-1 and *FgATG9* deletion mutant. Bar = 10 μm. (D) Accumulation of lipid droplets in mycelia of Δ*Fgatg9* mutant after 48 h incubation in CM. LipidTOX is a dye that stains lipids. Fluorescence intensities of LipidTOX in PH-1 and Δ*Fgatg9* mutant are shown. Bar = 10 μm. (E) Degradation of lipid droplets in mycelia of Δ*Fgatg9* mutant after 18 h incubation in 1/10 DFM-C. Bar = 10 μm. Fluorescence intensities of LipidTOX in PH-1 and Δ*Fgatg9* mutant are shown.

To determine if FgAtg9 is required for the development of *F*. *graminearum*, PH-1, *FgATG9* deletion mutants (Δ*Fgatg9-2*, Δ*Fgatg9-3*) and Δ*Fgatg9-C* strains were grown on CM, PDA, SYM, MM, MM-N agar for 3 days. We found that Δ*Fgatg9* mutants grew slower than PH-1 and Δ*Fgatg9-C* in all of the five media (Fig 5; Table 1). Furthermore, the Δ*Fgatg9* deletion mutants displayed totally flattened mycelia both in CM and SYM agar (Figs 5A, 6A and 6B) compared with PH-1 and Δ*Fgatg9-C*, similar to the defects observed in *FgATG8* and *FgATG15* deletion mutants [30,31]. This clearly demonstrates that FgAtg9 is involved in vegetative growth and aerial hyphae development. However, microscopic observation of the hyphae morphology of wild type PH-1 and *FgATG9* deletion mutant are not significantly different (Fig 6C).

**Table 1:** Phenotypic characterization of Δ*Fgatg9* mutants.

Strain	Colony diameter (mm)^a^	Conidiation (×10^4^/mL)^b^	Germination(%)^c^	Disease index^d^	DON (mg)^e^
PH-1	62.68 ±1.78^f^	200.11±14.54	96.51±3.69	11.44±2.27	34.00±11.19
Δ*Fgatg9-2*	53.82 ±0.75*	180.00±13.45	97.35±1.65	3.22±1.02**	35.50±12.32
Δ*Fgatg9-3*	52.52 ±1.32*	176.00±31.51	96.70±1.46	3.89±0.69**	34.64±11.20
Δ*Fgatg9-C*	58.58 ±1.12	190.17±23.03	95.83±1.56	11.22±0.51	33.73±11.97

^a^Colony diameter was measured after incubating in CM agar plates for 3 days.

^b^Conidiation was measured by counting the number of conidia in CMC culture for 3 days.

^c^Germination was measured by the percentage of germinated conidia in CM liquid culture after incubating for 8 hours.

^d^Disease index was rated by the number of symptomatic spikelet 14 days after inoculation. At least 3 wheat heads were examined in each repeat.

^e^DON was determined in liquid trichothecene biosynthesis media (TBI) for 7 days at 28°C. Mycelia were dried and measured to quantify the fungal biomass.

^f^Mean and standard error were calculated from at least three independent experiments.

*Asterisk represents significant difference at p < 0.05 according to *t*-test.

** Double asterisks represent significant difference at p < 0.01 according to *t*-test.

### FgAtg9 is required for lipid droplet degradation in response to starvation

It was reported that Atg15 is important for lipolysis of autophagic vesicles in *S*. *cerevisiae* and *F*. *graminearum* [31,45]. The deletion of *FgATG15* also displays aerial hyphae defect. We reasoned that the defects in aerial hyphae development of *FgATG9* deletion mutants may be due to decreased transport and degradation of lipid droplet. The mobilization of storage lipid droplets in carbon-starved mycelia were investigated in a modified liquid DFM medium with NO_3_^-^ as the only nitrogen source [30]. First, we used LipidTOX Red to stain the mycelia of PH-1 and Δ*Fgatg9* mutant after cultivating in liquid CM for 2 days. Numerous lipid droplets were observed to have accumulated in both PH-1 and Δ*Fgatg9* mutant as evidenced by the fluorescence intensity of LipidTOX Red (Fig 6D). The result suggested that FgAtg9 does not affect the storage of lipid droplets. Next, we washed the mycelia with water and then transferred them to 1/10 DFM-C (carbon-starved) media for 18 hours. As a result, the Δ*Fgatg9* mutant retained most of the lipid droplets while the wild type PH-1 had significantly reduced lipid droplets to support the fungal metabolism (Fig 6E). These results indicate that deletion of *FgATG9* affects lipid droplet degradation in response to starvation.

### FgAtg9 is required for pathogenicity

**Fig 7:**
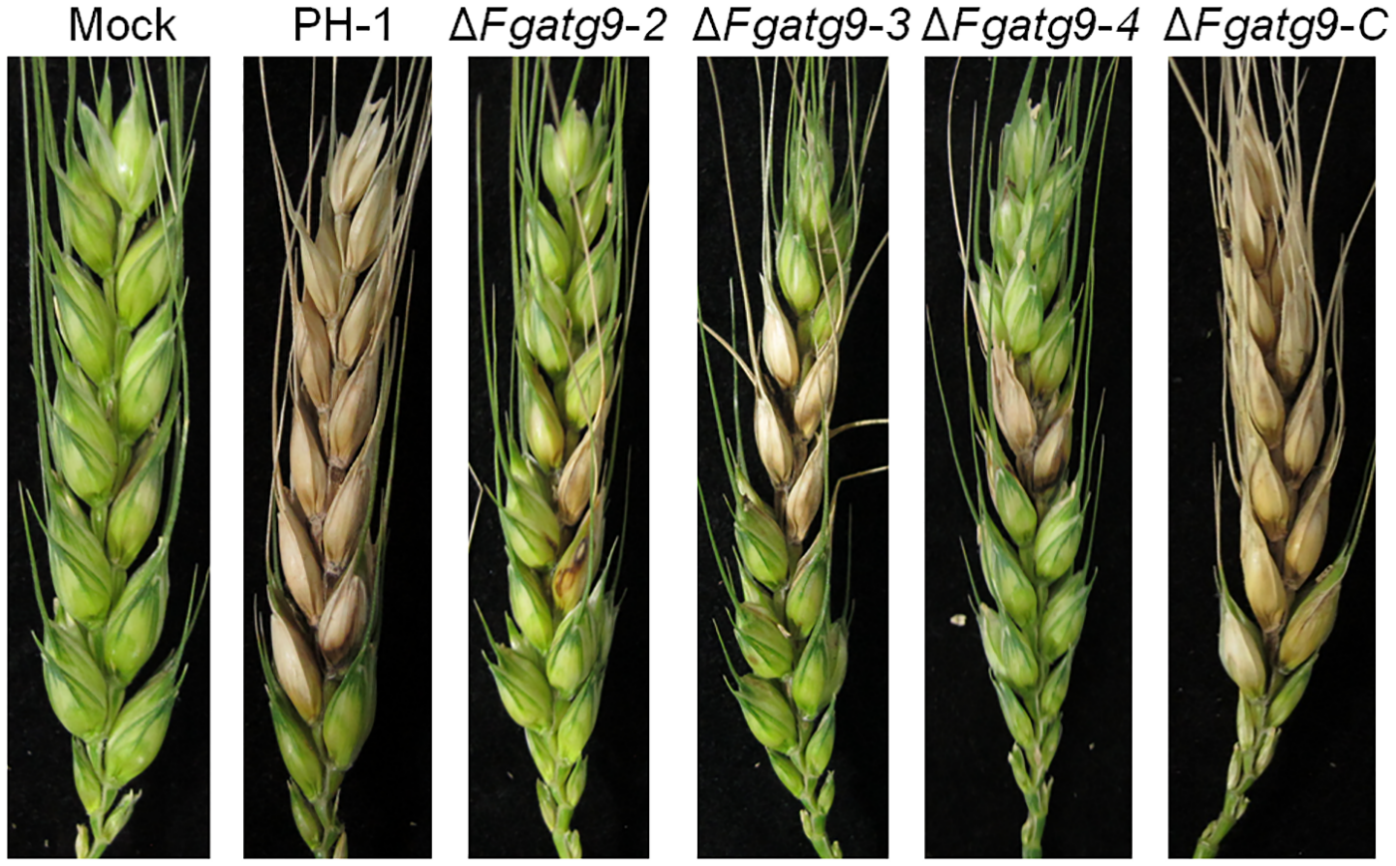
Infection of flowering wheat heads by *FgATG9* deletion mutants. Flowering wheat heads were inoculated with the wild type (PH-1) and Δ*Fgatg9* mutants. The infection assay was terminated after 14 days and pictures were taken to show the typical disease lesions of multiple experiments.

In infection assays with flowering wheat heads, the pathogenicity of Δ*Fgatg9* mutants significantly decreased in comparison with the wild type PH-1 (Fig 7; Table 1). The PH-1 and the complemented strain Δ*Fgatg9-C* caused typical head blight symptoms in the inoculated kernels which spread to other spikelets on the same heads at similar rates (Fig 7), whereas the blight symptoms caused by the Δ*Fgatg9* mutants spread to the nearby spikelets at much slower rate under the same condition (Fig 7), indicating reduced virulence in the Δ*Fgatg9* mutants.

### The sensitivity of Δ*Fgatg9* mutants to stress response

**Fig 8:**
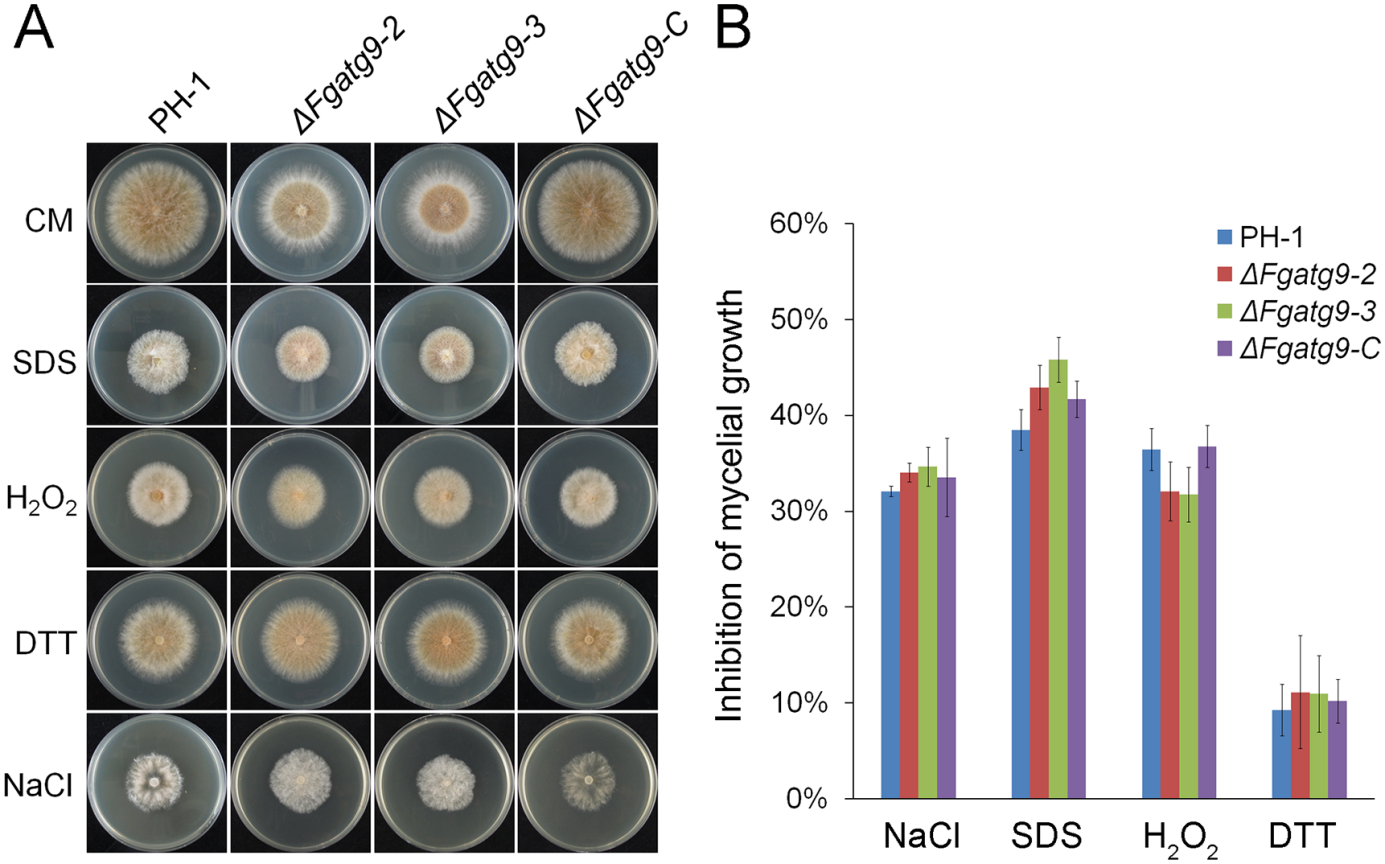
The sensitivity of PH-1, *FgATG9* deletion mutants and complemented strain to cytosolic membranes, oxidative, endoplasmic reticulum and osmotic stress agents. (A) The colony morphology of PH-1, Δ*Fgatg9* and Δ*Fgatg9-C* strains grown on CM plates with different stress agents (0.01% SDS, 0.03% H_2_O_2_, 5 mM DTT and 1 M NaCl) for 3 days. (B) The percentage of the mycelial radial growth inhibition was quantified after incubation of the PH-1, Δ*Fgatg9* and Δ*Fgatg9-C* on CM plates with or without stress agents for 3 days.

Autophagy is activated for cell survival when endoplasmic reticulum (ER) is stressed in mammalian cells and the same process is also involved in stress responses in plants [46,47]. Oxidative stress also induces autophagy [48]. However, whether Atg9 is involved in these various types of stress responses is still unknown in plant pathogenic fungi. To determine if FgAtg9 is required for response to plasma membrane (SDS), oxidative (H_2_O_2_), endoplasmic reticulum (DTT) and osmotic (NaCl) stresses, we investigated the vegetative growth of the Δ*Fgatg9* mutants in the presence of SDS, H_2_O_2_, DTT, and NaCl in CM media, and found that the Δ*Fgatg9* mutants were only slightly more sensitive to cytosolic membranes, endoplasmic reticulum and osmotic stress agents, but slightly less sensitivity to H_2_O_2_, an oxidative stress agent (Fig 8A and 8B). These results suggest that FgAtg9 is dispensable for stress response in *F*. *graminearum*.

### FgAtg9 is not required for sexual and asexual reproduction in *F*. *graminearum*

Conidia and ascospores of *F*. *graminearum* are believed to be the main inocula infecting flowering wheat heads [49,50]. We therefore inoculated the strains on carboxymethylcellulose (CMC) medium to harvest their conidia for comparison. We found that the conidiation of the Δ*Fgatg9* mutants showed little difference from that of the wild type PH-1 (Table 1). Similarly, the conidial germination of the Δ*Fgatg9* mutants was also the same as the wild type and the complemented strain Δ*Fgatg9-C* (Table 1). Furthermore, the perithecia and ascospores produced by the Δ*Fgatg9* mutants were similar, in morphology to those produced by the PH-1 and Δ*Fgatg9-C* (S5 Fig). Therefore, we conclude that FgAtg9 is not important for both sexual and asexual reproductions in *F*. *graminearum*.

### Heterologous expression of *MoATG9* rescues the defects of *FgATG9* deletion mutant

**Fig 9:**
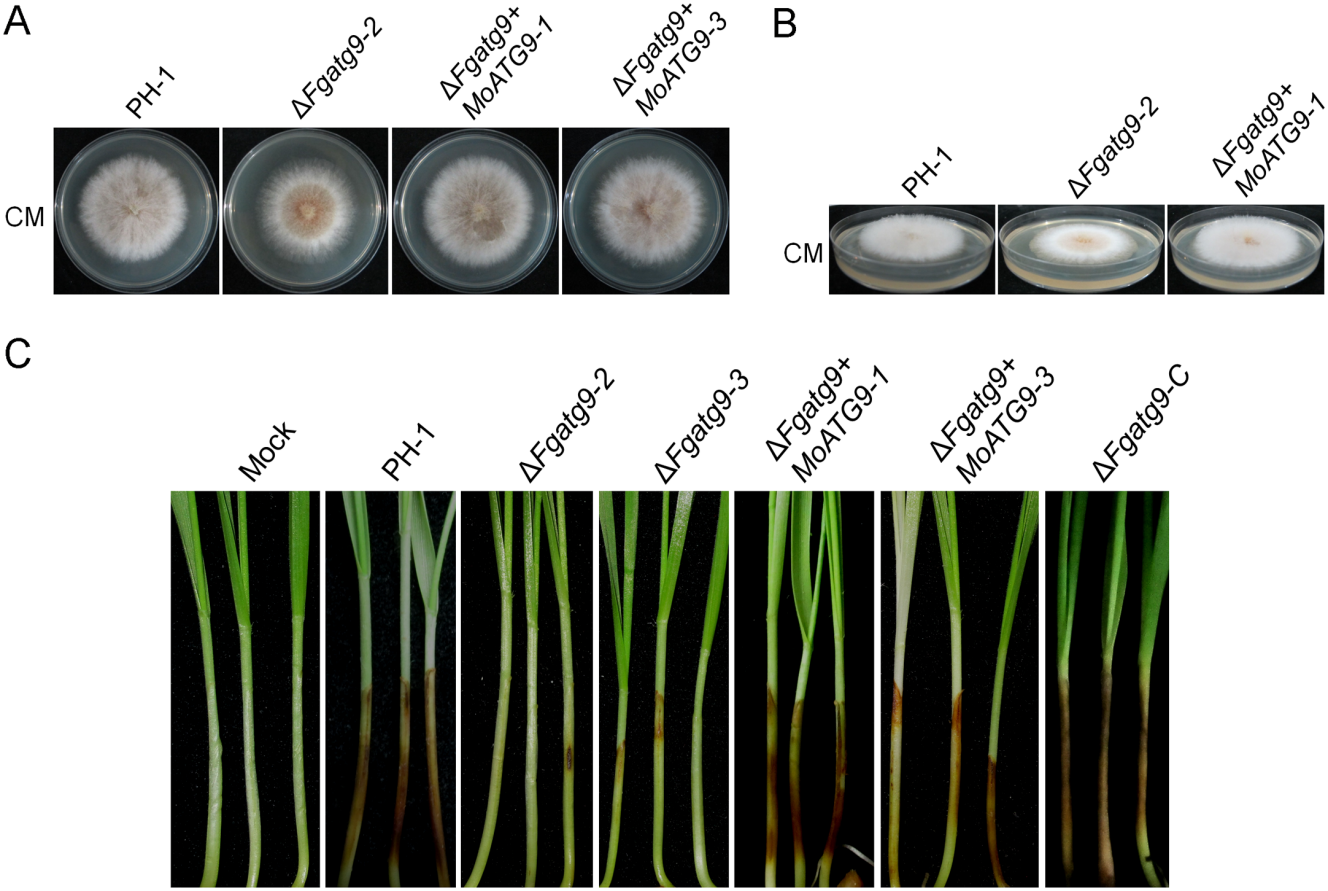
Heterologous expression of *MoATG9* rescues the defects of *FgATG9* deletion mutant. (A) Colony morphology of the Δ*Fgatg9* mutant and the heterologous complemented strains in CM medium. (B) The aerial hyphae morphology of Δ*Fgatg9* mutant and heterologous complemented strains in CM medium. (C) *MoATG9* restored the pathogenicity defect of Δ*Fgatg9* mutant.

MoAtg9 and FgAtg9 show a close relationship according to the conserved transmembrane domains and phylogenetic tree analysis. To test if MoAtg9 can functionally replace FgAtg9, we introduced *MoATG9* gene with its native promoter into the Δ*Fgatg9* deletion mutant and the resulting transformants showed that *MoATG9* expression successfully rescued the defect in vegetative growth of the *FgATG9* deletion mutant (Fig 9A), and displayed a normal aerial hyphae similar to the wild type PH-1 (Fig 9B). To determine whether it could also rescue the pathogenicity defect of the mutant, we inoculated wheat coleoptiles with the Δ*Fgatg9+MoATG9* transformants. Like the wild type PH-1, the Δ*Fgatg9+MoATG9* transformants caused severe disease lesions on wheat coleoptiles while the Δ*Fgatg9* strains caused little disease symptoms (Fig 9C). Taken together, these results indicate that MoAtg9 can functionally complement the observed defects in Δ*Fgatg9* mutant, suggesting a conserved function of Atg9 during the evolution of filamentous fungi particularly *F*. *graminearum* and *M*. *oryzae*.

## Discussion

Autophagy plays important roles during development and disease conditions in eukaryotes as well as pathogenesis of all pathogenic eukaryotes [16]. One fundamental question in the autophagy field is how the autophagosomes are formed and the recycling of cellular elements to ensure survival under stress conditions [16]. Atg9 is the sole multi-spanning membrane protein of the autophagy-related proteins. However, the functions of *ATG9* gene homologues are still unclear in filamentous fungi. Previous studies indicated that Rab GTPases, clathrin and/or adaptor proteins, and the retromer complex are all important for Atg9-mediated autophagy in mammalian cells [39,51–54]. Here, we demonstrate for the first time that the small GTPase FgRab7 is required for FgAtg9 trafficking, which is essential for autophagy, development, and pathogenicity in *F*. *graminearum*.

Recent studies demonstrated that the N-terminal cytoplasmic domain of Atg9A, which binds AP-2 for trafficking through the recycling endosomes, is required for autophagosome formation [55]. FgAtg9 has five conserved transmembrane domains, and we speculate that FgAtg9 may traverse the plasma membrane or endosomal compartments and contribute to the formation of autophagosomes. Our data show that GFP-FgAtg9 mainly localizes to the late endosomes and TGN under both nutrient-rich and nitrogen starvation conditions, respectively. Atg9-containing compartments are a source of membranes for the formation and/or expansion of autophagosomes [56], in support of the contention that late endosomes and TGN may be the original sources of autophagosomal membranes in *F*. *graminearum*. Furthermore, we found that disruption of the actin cytoskeleton results in restricted movement of FgAtg9, consistent with a previous report that the actin cytoskeleton is important for anterograde delivery of Atg9 to the PAS [23].

Atg9 has been shown to be essential for autophagy in yeast but displays mild autophagy phenotype in higher plants [20,47]. In this study, we demonstrate that FgAtg9 is an essential component of the core machinery for the formation of autophagosomes during autophagy. Previous studies indicated that Rab7 is required for the maturation of autophagosomes [37] and that FgMon1 serves as a guanine nucleotide exchange factor for FgRab7 and is also important for autophagy [57]. Atg9 cycles between the TGN and Rab7-positive endosomes in mammalian cells [40]. However, whether Rab7 is required for the cycling of Atg9 is still unknown. In this study, we established that FgAtg9 localizes to FgRab7-positive late endosomes and it is in close association with FgRab7 in an *in vivo* Co-IP assay, and requires the small GTPase FgRab7 for its trafficking, suggesting that FgRab7-mediated trafficking is essential for the function of FgAtg9. However, we do not have the evidence to show whether this regulation is a direct one.

Defects in autophagy genes in filamentous fungi can influence morphogenesis and development under nutrient-rich condition. For example, *ATG1*, *ATG8* and *ATG15* deletion mutants consistently show reduced number of aerial hyphae [31,58]. FgAtg9 is important for normal growth and pathogenicity of *F*. *graminearum* which is consistent with a recent study [29]. However, we demonstrated that FgAtg9 is not required for conidiation on CMC medium, contrary to a previous report that FgAtg9 is important for sporulation in mung bean liquid (MBL) cultures [29], possibly due to different nutritional conditions. Taken together, our findings support the contention that the autophagy pathway is required for cell differentiation and development of filamentous fungi in nutrient-rich media. However, a previous study in the filamentous yeast *Candida albicans* indicated that autophagy disruption due to *ATG9* deletion does not affect hyphal differentiation or formation of chlamydospores [59].

*M*. *oryzae* is another filamentous plant pathogen which causes rice blast disease and *MoATG9* is required for autophagy and plays important roles during the fungal foliar infection process in *M*. *oryzae* [26,27]. Our phylogenetic analysis and heterologous functional complementary experiments both suggest that Atg9 is highly conserved between *F*. *graminearum* and *M*. *oryzae*, although the two plant pathogens have different hosts. Autophagy is required for spore collapse (cell death) during host infection in *M*. *oryzae* [25]. *FgATG15* deletion mutants are defective in conidiation [31], but FgAtg9 shows normal conidia development, indicating that FgAtg9 is not important for asexual development.

DON as one of the secondary metabolites produced by *F*. *graminearum* contaminates cereal grains [7]. Previous studies suggest that some *ATG* genes such as *FgATG2*, *FgATG8*, and *FgATG15* are involved in DON production [29]. However, we demonstrated here that FgAtg9 is not important for the production of DON, suggesting that different *ATG* genes affect DON production in different ways.

In summary, we identified an autophagy-related protein (FgAtg9) in *F*. *graminearum* in this study and showed that FgRab7-mediated FgAtg9 trafficking is essential for autophagy and that FgAtg9 plays important roles in vegetative growth, aerial hyphae development, lipid metabolism and pathogenicity in *F*. *graminearum*. These results will expand our understanding of the relationship between membrane trafficking and the autophagy-dependent development and pathogenicity in plant fungal pathogens.

## Materials and methods

### Strains and culture conditions

Wild type (PH-1) and mutant strains used in this study are listed in S1 Table. PH-1 and all mutants were grown and evaluated by culturing the strains on complete medium (CM), potato dextrose agar medium (PDA), starch yeast medium (SYM), minimal media (MM) or minimal media for nitrogen starvation (MM-N) at 28°C for 3 days [12]. Sexual reproduction was assayed on carrot agar medium according to a previous report [60]. Conidiation was measured as previously reported [61]. For conidia germination assays, freshly harvested macroconidia were suspended in CM for 4 h with gentle agitation [62]. Conidia of PH-1 and the mutants were observed using an Olympus BX51 Microscope and Nikon A1R Laser Scanning Confocal Microscope. Aerial hyphae of the wild type and the Δ*Fgatg9* mutant were photographed after cultivating on CM medium plate for 3 days or in test tubes containing 5 ml of CM agar for 5 days.

### *FgATG9* gene disruption

*F*. *graminearum* protoplast preparation and fungal transformation were performed following standard protocols [63]. The split-marker approach [64] was used to generate gene replacement construct for the *FgATG9* gene. The primers used to amplify the flanking sequences for each gene are listed in S2 Table. Three knockout candidates were further verified by Southern blot with the Digoxigenin High Prime DNA Labeling and Detection Starter Kit I (Roche).

### Construction of mCherry-FgAtg9, ToxA-GFP-FgAtg9 and Flag-FgRab7 fusion vectors and complementation

The ToxA-GFP-FgAtg9 fusion vector was constructed by amplification of 3100-bp FgAtg9 coding sequence and 3’UTR using the primers FgATG9GF and FgATG9OR-WF-EcoRI (listed in S2 Table). ToxA-WF-XhoI and GFPR primers were used to amplify the ToxA-GFP fragment from the pCT74 plasmid [65] and the PCR products were cloned into pKNT vector using One Step Cloning Kit (Vazyme Biotech Co., Ltd) and verified by sequence analysis. For mCherry-FgAtg9 fusion vector, FgATG9ZF-WF and FgATG9ZR primers were used to amplify the native promoter from the genomic DNA of the PH-1 and tagged with mCherry at the N-terminus of the FgAtg9 coding sequence. For Flag-FgRab7 fusion vector, FgRab7-ZF-IP and FgRab7-ZR-IP-Flag were used to amplify the native promoter and Flag sequence, FgRab7-OF-IP and FgRab7-GR-IP were used to amplify the coding sequence from the genomic DNA of PH-1, the PCR products were cloned into pKNT vector using One Step Cloning Kit and verified by sequence analysis. MoATG9CF and MoATG9CR were used to amplify the native promoter and coding sequence from the genomic DNA of Guy11. The products were finally transformed into the Δ*Fgatg9* mutant or wild type PH-1 protoplasts. Transformants were screened by PCR with primer pairs (S2 Table) or further confirmed by fluorescence signal.

### Plant infection and DON production assays

Infection assays on flowering wheat heads were conducted as previously described [12] and the developed symptoms were observed 14 days after inoculation. For wheat coleoptiles infection assays, 4×10^4^/ml conidial suspension were inoculated and symptoms observed 8 days after inoculation. For DON production assays, all strains were grown in liquid trichothecene biosynthesis media (TBI) at 28°C for 7 days, the liquid and mycelia were then collected, respectively. The collected liquid was used for enzyme linked immunosorbent assay (ELASE) whereas the mycelia were dried and measured to quantify the fungal biomass.

### Lipid staining

*F*. *graminearum* PH-1 and the Δ*Fgatg9-2* mutant were grown in CMC for 3 days to generate conidia. The conidia obtained were collected and cultivated in liquid CM medium with 4×10^4^/ml conidial suspension at 28°C for 2 days. Mycelia were harvested and washed twice with water and inoculated in 1/10 DFM-C for starvation for about 18 h [30]. Lipid droplets from the mycelia were visualized by staining with HCS LipidTox Red (Invitrogen) at 0 h and 18 h after under starvation.

### Chemical inhibitors and live cell imaging of *F*. *graminearum*

Nocodazole (Sigma, final concentration 100 μM), LatA (latrunculin A, Sigma, final concentration 10 μM) and CFW (Calcofluor White, Sigma, final concentration 10 μg/ml) were used according to our previously reported [9,12]. Nikon A1R laser scanning confocal microscope system was used for live cell fluorescence imaging (Nikon, Japan). Elapsed time is indicated in seconds. CFW excitation used 405 nm light (Em. 452/45 nm), GFP excitation was performed with 488 nm light (Em. 525/40 nm), HCS LipidTox Redor mCherry excitation was performed with 561 nm light (Em. 607/36 nm).

### Autophagy induction, western blot and transmission electron microscopy observation

For autophagy assay, 4×10^4^/ml conidial suspension were cultured in liquid CM medium for 16 h. Mycelia were harvested and washed twice with water and then transferred to nitrogen-limiting medium (MM-N) in the presence of 2 mM PMSF for 8 h. GFP-FgAtg8 were visualized and total proteins were extracted at 0 h and 8 h after starvation. For immunoblot analysis of GFP-fusion-proteins from cellular extracts, equal concentrations of total proteins were isolated and analyzed by immunoblot detection with the anti-GFP (GFP-Tag Mouse mAb, Abmart, China) and anti-actin antibodies (Actin-Tag Mouse mAb, Abmart, China) following a previous report [66]. For immunoprecipitation, total proteins were isolated and incubated with 30 μL of GFP-Trap_A beads according to the manufacturer’s instructions. Proteins eluted from the GFP-Trap_A beads were analyzed by immunoblot detection with an anti-Flag antibody (Flag-Tag Mouse mAb, Abmart, China) and anti-GFP antibody. Transmission electron microscopy was carried out as previously described to observe the autophagic bodies [66].

## Supporting information

S1 Fig Conserved transmembrane domains and phylogenetic analysis of Atg9 in different species. (A) FgAtg9 has five conserved transmembrane domains. (B) Phylogenetic tree of Atg9 proteins from different organisms. A neighbor-joining tree is shown based on the amino acid sequences of representative fungi. The numbers at nodes represent the percentage of their occurrence in 10,000 bootstrap replicates.
(TIF)

S2 Fig The subcellular localization of GFP-FgAtg9. (A) The Western blot of GFP-FgAtg9 strains with GFP antibody, the band size of GFP-FgAtg9 protein is 129.2 kDa. (B-C) Expression of GFP-FgAtg9 fusion protein in mycelia at different time points (0, 4 h, 8 h) during conidial germination.
(TIF)

S3 Fig The kymographs of GFP-FgAtg9 from S2, S3 and S4 Videos respectively. GFP-FgAtg9 treated with DMSO (control), LatrunculinA (an actin cytoskeletons inhibitor) and Nocodazole (a microtubule-destabilizing agent) respectively. Time duration is 58.13 s.
(TIF)

S4 Fig Southern blot analysis to confirm *FgATG9* gene deletion. (A) The scheme of split-marker approach based on the targeted gene replacement of *FgATG9* by *hph* gene. Genomic DNAs were extracted from PH-1 and putative transformants. (B) Targeted gene deletion of *FgATG9*. *Bam*H I(B) digested DNAs showed a 4.46 kb band in the PH-1 and a 2.87 kb band in the mutants, both bands were present in the complemented strain.
(TIF)

S5 Fig Perithecium and ascospore formation of PH-1, Δ*Fgatg9* mutants and complemented strain. (A) The conidial morphology of Δ*Fgatg9* mutant compare with PH-1. Bar = 10 μm. (B) Perithecium formation of indicated strains on carrot agar plates. Bar = 500 μm. (C) The ascospore released from the perithecia of indicated strains. Bar = 100 μm.
(TIF)

S1 Table Wild type (PH-1) and mutant strains of the fungi used in this study. (DOC)

S2 Table PCR primers used in this study. (DOCX)

S1 Video Mobility of GFP-FgAtg9 in nutrient-rich medium. (AVI)

S2 Video Mobility of GFP-FgAtg9 after treatment with DMSO. (AVI)

S3 Video Mobility of GFP-FgAtg9 after treatment with Latrunculin A. (AVI)

S4 Video Mobility of GFP-FgAtg9 after treatment with Nocodazole. (AVI)

S5 Video Mobility of GFP-FgAtg9 under nitrogen starvation condition. (AVI)

S6 Video Mobility of GFP-FgAtg9 in Δ*Fgrab7* mutant under nutrient-rich condition. (AVI)

S7 Video Mobility of GFP-FgAtg9 in Δ*Fgrab7* mutant under nitrogen starvation condition. (AVI)
