# Supplementary figures and images for: Small GTPase Rab7-mediated FgAtg9 trafficking is essential for autophagy-dependent development and pathogenicity in Fusarium graminearum
Source: PLoS Genet. 2018 Jul 25;14(7):e1007546. doi: 10.1371/journal.pgen.1007546 (PMC6078321; doi:10.1371/journal.pgen.1007546)

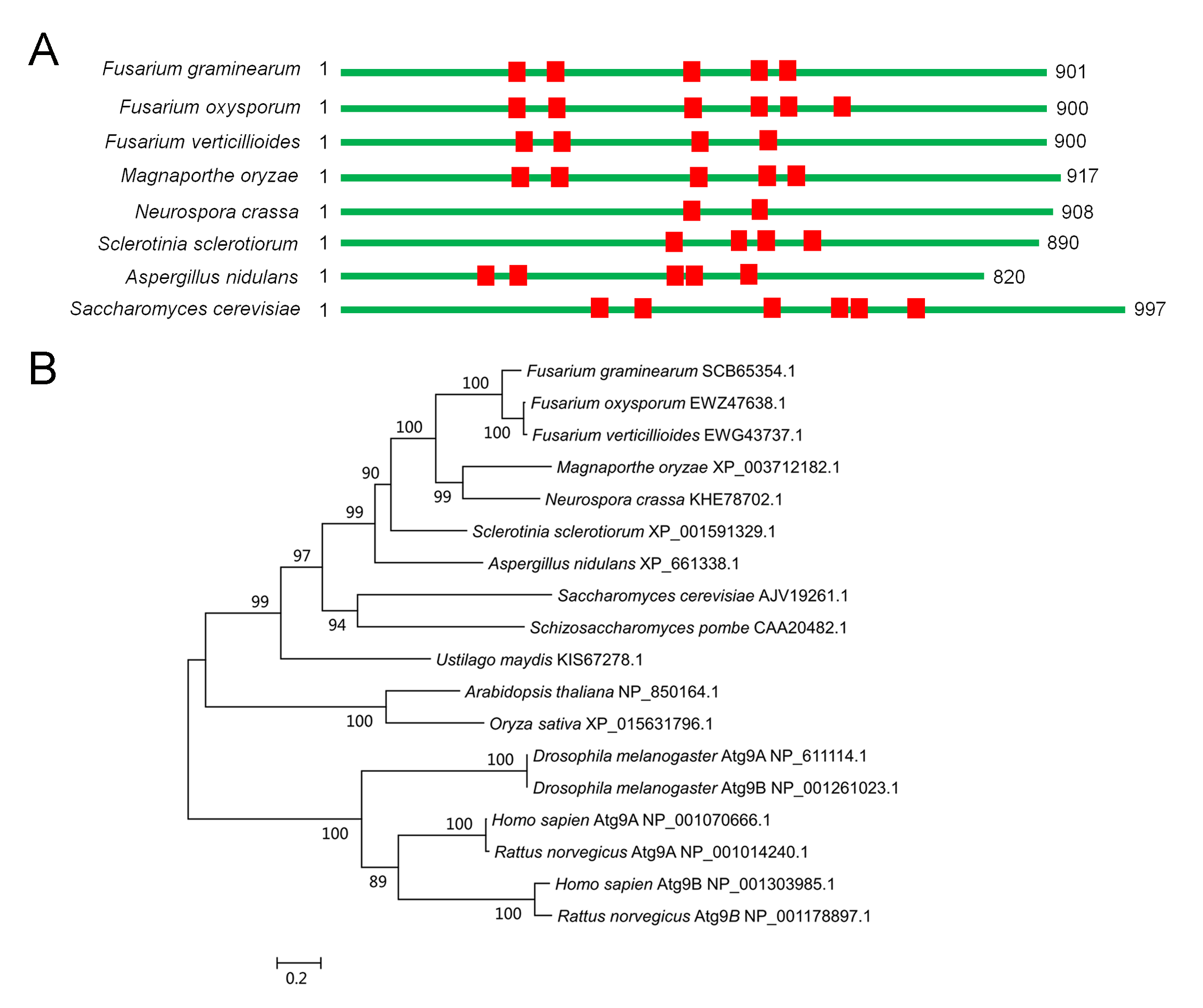

Supplement: S1 Fig — (A) FgAtg9 has five conserved transmembrane domains. (B) Phylogenetic tree of Atg9 proteins from different organisms. A neighbor-joining tree is shown based on the amino acid sequences of representative fungi. The numbers at nodes represent the percentage of their occurrence in 10,000 bootstrap replicates. (TIF) [file pgen.1007546.s001.tif]

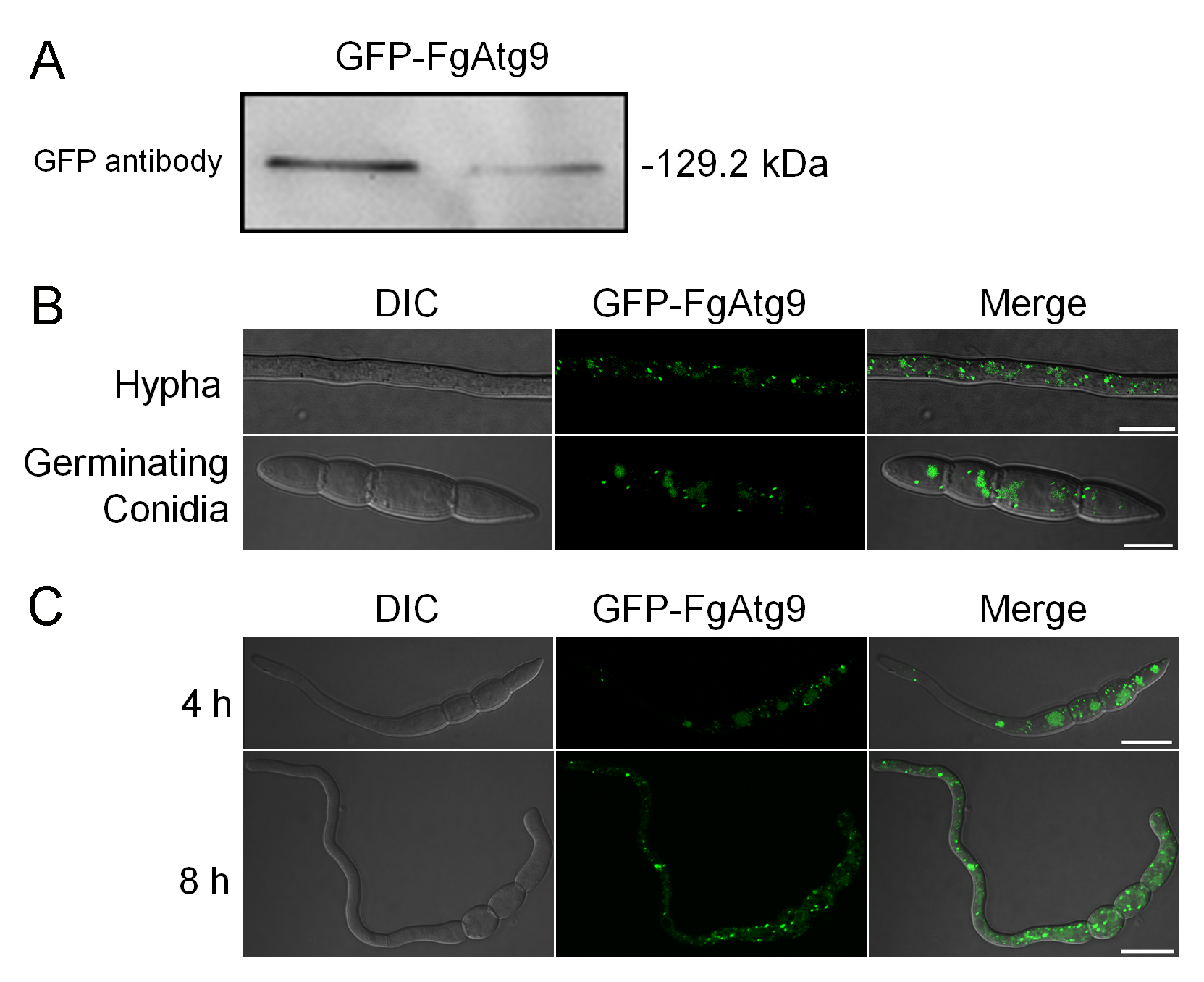

Supplement: S2 Fig — (A) The Western blot of GFP-FgAtg9 strains with GFP antibody, the band size of GFP-FgAtg9 protein is 129.2 kDa. (B-C) Expression of GFP-FgAtg9 fusion protein in mycelia at different time points (0, 4 h, 8 h) during conidial germination. (TIF) [file pgen.1007546.s002.tif]

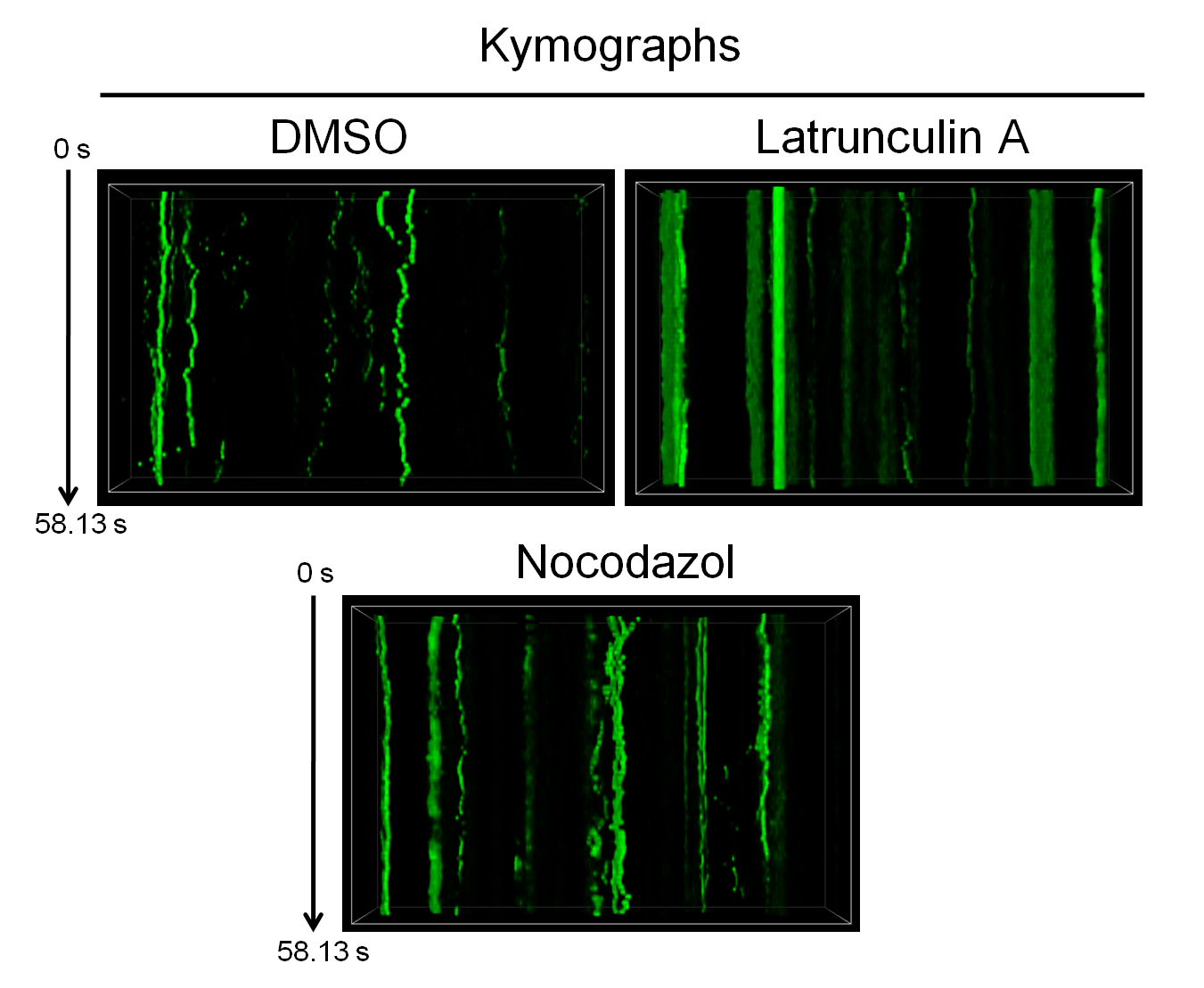

Supplement: S3 Fig — GFP-FgAtg9 treated with DMSO (control), LatrunculinA (an actin cytoskeletons inhibitor) and Nocodazole (a microtubule-destabilizing agent) respectively. Time duration is 58.13 s. (TIF) [file pgen.1007546.s003.tif]

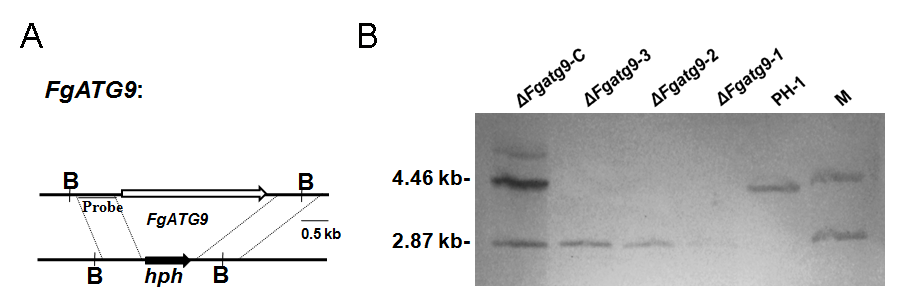

Supplement: S4 Fig — (A) The scheme of split-marker approach based on the targeted gene replacement of FgATG9 by hph gene. Genomic DNAs were extracted from PH-1 and putative transformants. (B) Targeted gene deletion of FgATG9. BamH I(B) digested DNAs showed a 4.46 kb band in the PH-1 and a 2.87 kb band in the mutants, both bands were present in the complemented strain. (TIF) [file pgen.1007546.s004.tif]

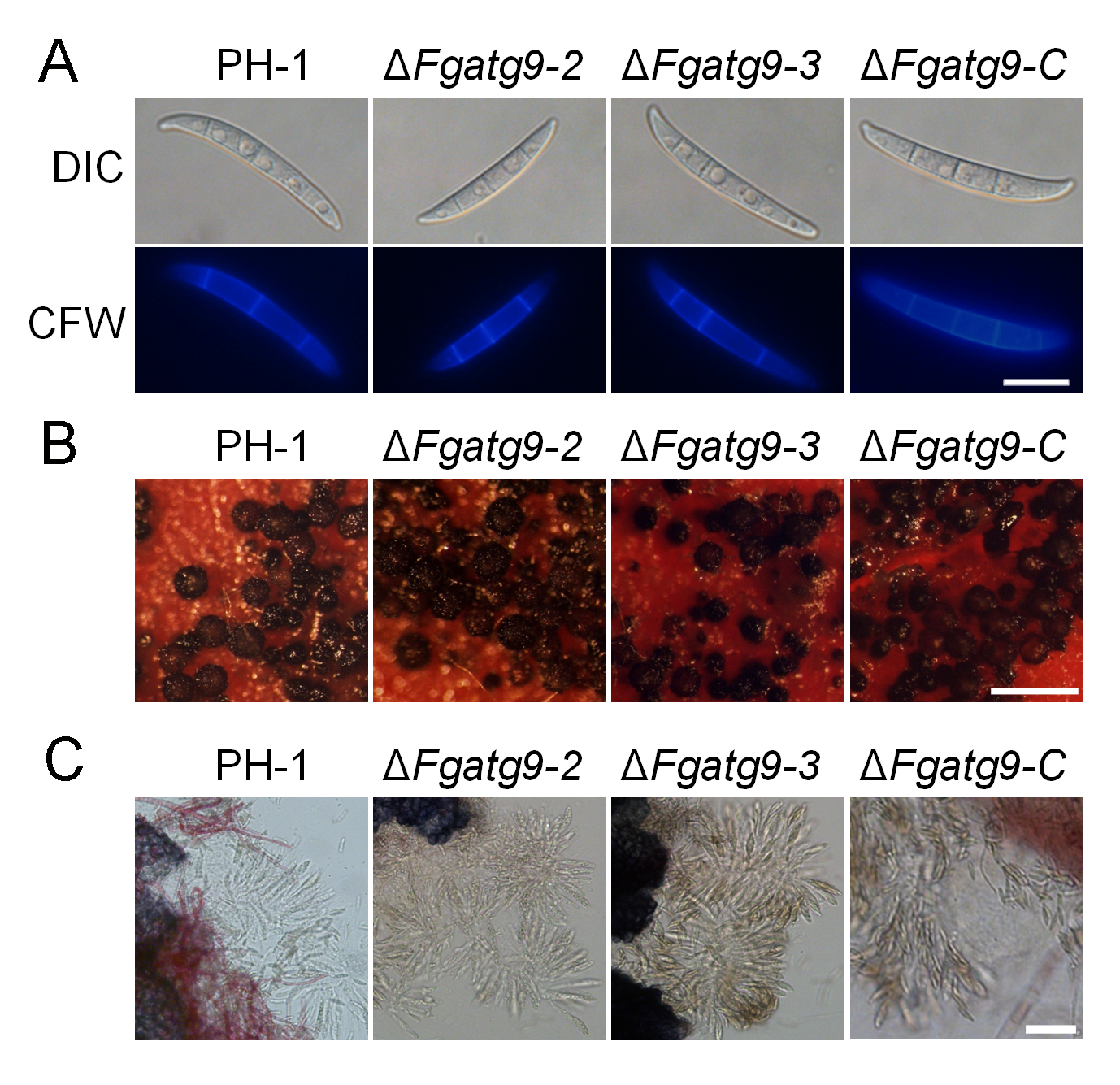

Supplement: S5 Fig — (A) The conidial morphology of ΔFgatg9 mutant compare with PH-1. Bar = 10 μm. (B) Perithecium formation of indicated strains on carrot agar plates. Bar = 500 μm. (C) The ascospore released from the perithecia of indicated strains. Bar = 100 μm. (TIF) [file pgen.1007546.s005.tif]
