# Supplementary material for: Small GTPase Rab7-mediated FgAtg9 trafficking is essential for autophagy-dependent development and pathogenicity in Fusarium graminearum
Source: PLoS Genet. 2018 Jul 25;14(7):e1007546. doi: 10.1371/journal.pgen.1007546 (PMC6078321; doi:10.1371/journal.pgen.1007546)
Supplement: S1 Table — (DOC) [file pgen.1007546.s006.doc]

**S1 Table Wild type (PH-1) and mutant strains of the fungi used in this study.**

| Strain | Genotype description | Reference |
| --- | --- | --- |
| PH-1 | Wild type |  |
| Δ*Fgatg8* | FGSG_10740 deletion mutant in PH-1 | This study |
| Δ*Fgatg9* | FGSG_13660 deletion mutant in PH-1 | This study |
| Δ*Fgatg9-C* | Δ*Fgatg9* strain expressing the FgAtg9 construct | This study |
| PH-1+GFP-FgAtg9+mCherry-FgRab52 | PH-1 strain expressing GFP-FgAtg9 and mCherry-FgRab52 constructs | This study |
| PH-1+GFP-FgAtg9+FgKex2-mCherry | PH-1 strain expressing GFP-FgAtg9 and FgKex2-mCherry constructs | This study |
| PH-1+GFP-FgAtg9+mCherry-FgRab6 | PH-1 strain expressing GFP-FgAtg9 and mCherry-FgRab6 constructs | This study |
| PH-1+GFP-FgAtg9+mCherry-FgRab7 | PH-1 strain expressing GFP-FgAtg9 and mCherry-FgRab7 constructs | This study |
| PH-1+GFP-FgAtg9+FgKar2-mCherry | PH-1 strain expressing GFP-FgAtg9 and FgKar2-mCherry constructs | This study |
| PH-1+GFP-FgAtg8 | PH-1 strain expressing GFP-FgAtg8 construct | This study |
| Δ*Fgatg9+*GFP-FgAtg8 | Δ*Fgatg9* strain expressing the GFP-FgAtg8 construct | This study |
| Δ*Fgatg9+*GFP-FgAtg9+mCherry-FgAtg8 | Δ*Fgatg9* strain expressing the GFP-FgAtg9 and mCherry-FgAtg8 constructs | This study |
| Δ*Fgrab51*+GFP-FgAtg9 | Δ*Fgrab51* strain expressing the GFP-FgAtg9 construct | This study |
| Δ*Fgrab7*+GFP-FgAtg9 | Δ*Fgrab7* strain expressing the GFP-FgAtg9 construct | This study |
| Δ*Fgrab8*+GFP-FgAtg9 | Δ*Fgrab8* strain expressing the GFP-FgAtg9 construct | This study |
| Δ*Fgatg9+*MoAtg9 | Δ*Fgatg9* strain expressing the MoAtg9 construct | This study |
| PH-1+GFP-FgAtg9+Flag-FgRab7 | PH-1 strain expressing GFP-FgAtg9 and Flag-FgRab7 constructs | This study |
| PH-1+GFP-FgAtg9 | PH-1 strain expressing GFP-FgAtg9 construct | This study |
| PH-1+Flag-FgRab7 | PH-1 strain expressing Flag-FgRab7 construct | This study |

Cuomo, C.A., Gueldener, U., Xu, J.R., Trail, F., Turgeon, B.G., Di Pietro, A.*, et al.* (2007) The Fusarium graminearum genome reveals a link between localized polymorphism and pathogen specialization. *Science* **317:** 1400-1402.
