# Supplementary material for: Small GTPase Rab7-mediated FgAtg9 trafficking is essential for autophagy-dependent development and pathogenicity in Fusarium graminearum
Source: PLoS Genet. 2018 Jul 25;14(7):e1007546. doi: 10.1371/journal.pgen.1007546 (PMC6078321; doi:10.1371/journal.pgen.1007546)
Supplement: S2 Table — (DOCX) [file pgen.1007546.s007.docx]

**S2 Table PCR primers used in this study**

| Primer | Sequence 5’-3’ | Description |
| --- | --- | --- |
| FgATG9AF | GTCTGAAGGAGCCATTGTGA | *FgATG9* deletion |
| FgATG9AR | TTGACCTCCACTAGCTCCAGCCAAGCCTCGGCGATGATGGAGAAT |  |
| FgATG9BF | GAATAGAGTAGATGCCGACCGCGGGTTACGACGGCGGTGAATCTA |  |
| FgATG9BR | AGGCACAAAGTCCCAGCA |  |
| HYG/F | GGCTTGGCTGGAGCTAGTGGAGGTCAA |  |
| HY/R | GTATTGACCGATTCCTTGCGGTCCGAA |  |
| YG/F | GATGTAGGAGGGCGTGGATATGTCCT |  |
| HYG/R | AACCCGCGGTCGGCATCTACTCTATTC |  |
| FgATG9OF | TAGCCTTCATGTCGGGTGC |  |
| FgATG9OR | CGGGTTTGCTGCGTAGTTAT |  |
| FgATG9UA | TGTTCTTCGACGTTTGTTCCA |  |
| H853 | GACAGACGTCGCGGTGAGTT |  |
| ToxA-WF-XhoI | GGGTACCGGGCCCCCCCTCGAGTGGAATCCATGGAGGAGTTC | Construction of GFP-FgAtg9 vector |
| GFPR | CTTGTACAGCTCGTCCATGC |  |
| FgATG9GF | GCATGGACGAGCTGTACAAGATGGCATCAAACATATTCTCCCGGATA |  |
| FgATG9OR-WF- EcoRI | CCCCCGGGCTGCAGGAATTCCCGGAGCTGACATCGACAC |  |
| FgATG9ZF-WF | GGGTACCGGGCCCCCCCTCGAGTTCTGAAACAGCGATGGTC | Amplification of FgAtg9 native promoter sequence |
| FgATG9ZR | TCCTCGCCCTTGCTCACCATCGCATATCTGTTGGCTCGAT |  |
| MoATG9CF | GGCTCCTCAGCACCTATT | Amplification of *MoATG9* gene |
| MoATG9CR | GTCCGCCTCGGGTATCTT |  |
| FgRab7-ZF-IP | GGGTACCGGGCCCCCCCTCGAGCTAGGCTATGCAAAGCAGC | Construction of Flag-FgRab7 vector |
| FgRab7-ZR-IP-Flag | TTTGTCGTCATCGTCTTTGTAGTCTTTGTCGTCATCGTCTTTGTAGTCTTTGTCGTCATCGTCTTTGTAGTCCATACCACCGGTGAATAATCGTTTGTAAA |  |
| FgRab7-OF-IP | CTACAAAGACGATGACGACAAAATGTCTTCTCGAAAGAAGGT |  |
| FgRab7-GR-IP | CCCCCGGGCTGCAGGAATTCCCTGATGGCTTCATCTTCC |  |
